# Do Terrorists Get the Attention They Want? Comparing Effects of Terrorism across Europe

**DOI:** 10.1093/poq/nfab046

**Published:** 2021-10-07

**Authors:** Enzo Nussio, Tobias Böhmelt, Vincenzo Bove

## Abstract

Terrorists aim at influencing audiences beyond their immediate victims, but can only achieve this if an attack receives sufficient public attention. Previous research shows that terrorism can affect public opinion, but these studies are mainly based on emblematic single cases and relate to varying measures of influence, which are difficult to compare. This research focuses on the first-order effect of terrorism: attention. To analyze whether terrorists get attention, we combine a quasi-experimental approach for causal identification with a comparative design. We compile data from Eurobarometer surveys and contrast responses of more than 80,000 individuals surveyed before and after five diverse Islamist attacks in Europe in 2013–2019. Attention to terrorism increases in all targeted countries, regardless of attack size. Yet, while all incidents raise attention to terrorism, only larger attacks exert a meaningful impact across Europe.

## Introduction

Terrorism poses a small risk of victimization but is a major source of public fear. By becoming a priority for policymakers and a core concern for many citizens in the Western world, terrorism can crucially shape democratic institutions, electoral behavior, and individual well-being (e.g., Huddy et al. 2005; Legewie 2013; Getmansky and Zeitzoff 2014; Balcells and Torrats-Espinosa 2018; Böhmelt, Bove, and Nussio 2019).

In this research note, we contribute to the growing literature on the consequences of terrorism. Existing quasi-experimental studies on this topic, comparing answers to surveys conducted right before and after attacks (Muñoz, Falcó-Gimeno, and Hernández 2020), offer high internal validity. Yet, as they frequently focus on single cases, their generalizability remains unclear. To address this limitation, we embed a quasi-experimental approach, allowing for strong causal identification, in a comparative design, which increases external validity, to answer a key question: do terrorists get the attention they want?

Surprisingly, few studies compare how terrorism affects public opinion across attacks. We compare effects across five attacks, which vary in their number of fatalities. While attack size is arguably related to impact (Rohner and Frey 2007), this relationship may not be as clear as conventional wisdom suggests. Small-scale events, like a knife attack in the Netherlands in 2004, can have substantial impact (Finseraas, Jakobsson, and Kotsadam 2011), while attacks with many more victims, like the 2016 Berlin truck attack, may be surprisingly less influential (Nussio 2020). In any case, as terrorism comes in different forms, it is difficult to measure its consequences by studying individual attacks.

Research on the consequences of terrorism has identified various effects on public attitudes like trust in government, migration, and security preferences (e.g., Panagopoulos 2006; Mondak and Hurwitz 2012; Brouard, Vasilopoulos, and Foucault 2018; Nussio, Bove, and Steele 2019; Helbling and Meierrieks 2020a), with important downstream implications for security regulations (Bove, Rivera, and Ruffa 2019; Bove, Böhmelt, and Nussio 2020), radicalization (Mitts 2019), and war-making (Hetherington and Suhay 2011). The reactions to terrorism uncovered by these studies are often assumed to be due to an accrued salience of threat perception, particularly through feelings of imminent danger. Yet, existing studies rarely focus on the necessary prerequisite of terrorism influence: attention (exceptions include Criado 2017; Zuijdewijn and Sciarone 2019).

Terrorist attacks aim at obtaining a “political or social objective through the intimidation of a large audience beyond that of the immediate victims” (Enders, Sandler, and Gaibulloev 2011, p. 321). As a result, whether terrorism has an effect largely depends on an audience’s reception of it. At the same time, the increase in attention for terrorism can displace attention from other key concerns. Given the limited carrying capacity of the audience agenda, there is inevitable competition among rival issues such as taxation, security, or immigration. As such, the rise of one issue may result in the fall of another (Zhu 1992). In particular, so-called “killer issues” can displace other issues (Brosius and Kepplinger 1995). Terrorist attacks possess some of the main attributes of such killer issues, such as threatening personal consequences, symbolic value, and change in knowledge (Brosius and Kepplinger 1995). Simultaneously, by producing an immediate shock in media attention, terrorism has a greater agenda-setting potential compared with issues that attract less media attention (Geiß 2019). By focusing on the extent to which audiences view terrorism as an important issue in the aftermath of an attack, we thus also contribute to research on agenda-setting (Brosius and Kepplinger 1995; Geiß 2019) and its consequences for policymaking (Hetherington and Suhay 2011; Bove, Böhmelt, and Nussio 2020; Helbling and Meierrieks 2020b).

Empirically, we examine the perceptions of more than 80,000 individuals across 32 European countries from 2013 to 2019 in response to five terrorist attacks. Only Islamist terrorist attacks satisfy our design criteria (elaborated in the next section), likely because they are the most dominant type of recent terrorism in the Western world. We focus exclusively on Eurobarometer surveys to avoid confounding the effect of the attacks with differences in surveying procedures and to increase measurement comparability. We compare individuals surveyed right before the attack with individuals surveyed immediately after. We use questions related to whether people mention terrorism as one of the two most important issues facing their country (as phrased in the Eurobarometer questionnaires), treating positive answers as an indication of attention to terrorism.

When aggregating the five attacks, we find significant and causal effects as people’s attention to terrorism increases by about 2–3 percentage points across Europe. However, effects within targeted countries are larger, with the size of the impact ranging between 11 and 35 percentage points. Across the whole of Europe, effects vary between 0 and 7 percentage points, with the largest impact being associated with the November 2015 attack on the Bataclan theater and smaller incidents negligibly affecting the attention to terrorism.

## Design

We compare individuals interviewed before and after five terrorist attacks in 2013–2019 by looking for incidents that occurred during the field period of a respective Eurobarometer wave. Terrorist attacks are rare events, and surveys with comparable designs fielded while a terrorist event occurs are even less readily available. Therefore, identifying five quasi-experimental situations with comparable evidence represents a unique contribution. To identify attacks, we compared the dates of lethal terrorist attacks in Europe using the Global Terrorism Database (GTD) with the survey dates of the Eurobarometer in the post-9/11 period. The five identified attacks are thus an “as-if random” selection of terrorism in Europe, as surveying periods are not related to terrorism.^1^ All five attacks were Islamist inspired: London (2013), Paris and Saint Denis (2015), Manchester (2017), Carcassonne (2018), and Utrecht (2019). The Supplementary Material provides an overview of all five incidents.

The identification strategy of our quasi-experimental design assumes that the timing of the interviews must have occurred randomly. We further assume that there should be no other time-varying influences coinciding with the observation period, which are systematically related to public opinion. All five events represent exogenous stimuli that randomly separate control and treatment groups, which lowers the risk of “alternative trends affecting potential changes” (Nussio, Bove, and Steele, 2019, p. 4). The treatment is assigned as if randomly, and by implementing Muñoz et al.’s (2020) best practices, we minimize the impact of other influences. The small difference in time and the absence of other notable events suggest that the two assumptions are met.

We merged all Eurobarometer datasets with the individual as the unit of analysis and eventually obtained 83,769 cases (before accounting for missing values) for the comparisons based on 3 days before/after. The sample size differs for before/after comparisons based on 1 and 5 days, respectively. That is, we distinguish between 1, 3, and 5 days for the before-after comparison of individuals’ attention to terrorism. These thresholds are based on Balcells and Torrats-Espinosa (2018) and acknowledge that it may take up to 24 hours until even highly salient news diffuses throughout the population (Rogers 2000); at the same time, effects may decrease and eventually disappear. Our thresholds are also largely in line with Geiß (2019), who finds that public responses to media impulses tend to peak around seven days, with television news wearing out more quickly. We analyze symmetric samples, and we disregard those who were interviewed before/after the specific thresholds. The main reasons for this approach are balancing and consistency across attacks, given that not all attacks allow for longer observation windows. In addition, short-range time windows before and after the attack minimize the possibility of other events driving the estimated effects. Note that we focus on first-order effects and, therefore, do not examine second-order “echo effects”.

The dependent variable, *Attention to Terrorism*, is constructed using one consistently worded item: “[w]hat do you think are the two most important issues facing (OUR COUNTRY) at the moment?” If individuals named terrorism as one of the two most important issues, *Attention to Terrorism* receives a value of 1 (otherwise 0), which was the case for 8 percent of the sample. While this may not be the primary goal of perpetrators, it is an indication of their success at grabbing attention. Our main explanatory variable, *Treatment*, receives a value of 1 if individuals were interviewed after an attack (about 46.3 percent in our sample), and 0 otherwise. Under the assumptions discussed above, any effect we identify can be interpreted as the average causal effect of the treatment on *Attention to Terrorism*.

**Figure 1.**
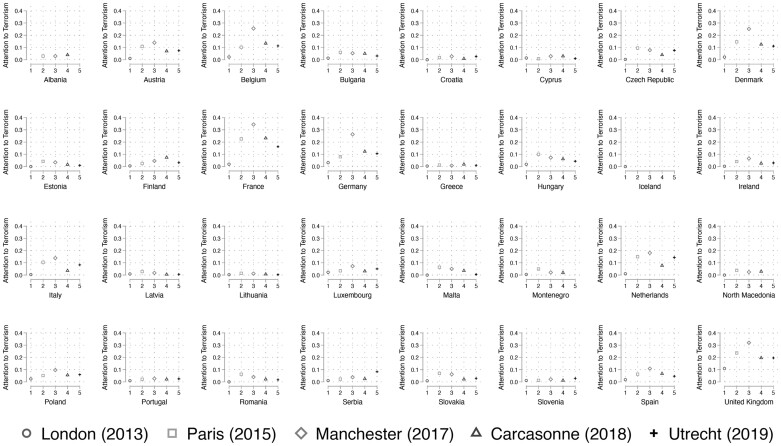
**Level of *Attention to Terrorism* by country.** Graph displays mean values aggregated to the country level.

Figure 1 gives an overview of the outcome variable. We focus on the proportion of respondents that indicated whether terrorism was one of the two most important issues affecting their country. For instance, almost 40 percent of the French sample responded affirmatively to this question in 2017. In general, figure 1 emphasizes that there is variation in terrorism attention both within and across countries. The overall attention to terrorism hovers near 0 in countries such as Slovenia, but is more strongly pronounced in others, including Denmark, France, Germany, and the United Kingdom. Interestingly, these countries differ in their experience with major attacks. We cover states that are diverse in terms of size, economic standing, culture, and ethnic composition. As we control for those and other potential influences, the findings we obtain cannot be attributed to one single state or a spurious influence.

We control for a series of individual-level characteristics, which may also be related to public opinion about terrorism (see Supplementary Material). Imbalance among these control variables is unlikely to affect our results (Supplementary Material, table S8). Given the hierarchical nature of our data (individuals nested in countries and years), we use hierarchical models and incorporate unit-level and survey-round intercepts to account for unobserved heterogeneity (see Supplementary Material).^2^ In the Supplementary Material, we also estimate logistic regression models with fixed effects (Supplementary Material, table S9). Additionally, we conducted falsification and placebo tests (Supplementary Material, table S11) and a selection test based on Oster (2019) (Supplementary Material, figure S1). Finally, we include individuals interviewed on the day of an attack (Supplementary Material, table S12) and consider wounded people in addition to fatalities (Supplementary Material, table S13).

## Analysis

### Overall Attack Effects on Attention to Terrorism

**Figure 2.**
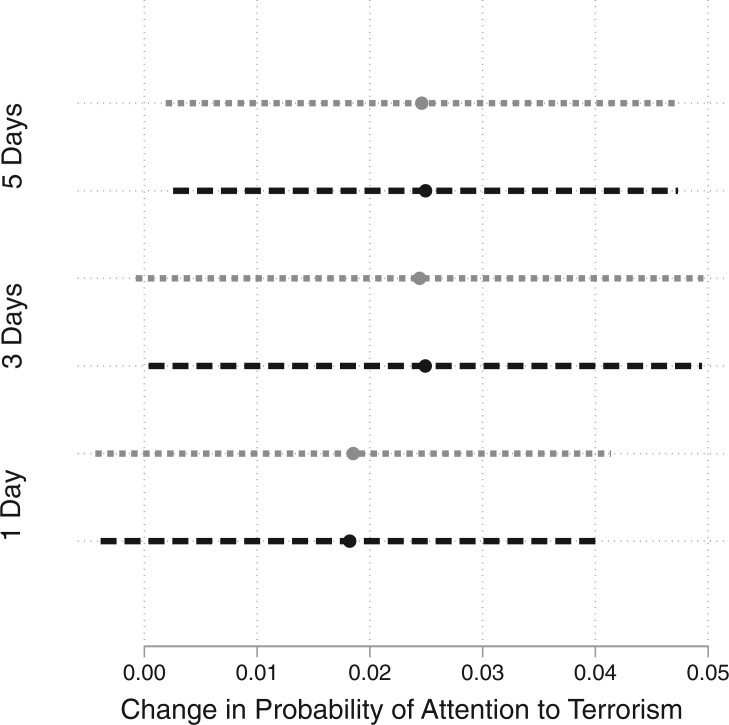
**Impact of attacks on *Attention to Terrorism*.** Point estimates marked by dots, 95 percent confidence intervals marked by horizontal bars. Black dots/lines represent models without controls; gray dots/lines represent models with control variables. Effects are calculated for different days before/after an attack. The Supplementary Material summarizes the *p*-values underlying this graph.

Figure 2 presents the findings of our aggregate analysis. The graph is based on six model estimations, where we distinguish between 1, 3, and 5 days for the before-after comparison of individuals’ attention to terrorism while including or excluding control variables (descriptive statistics of all variables, Supplementary Material, table S1). These models pool all five incidents that are covered in our data (regression models underlying the graphs, Supplementary Material, tables S2–S7). The point estimates in figure 2 capture the treatment—whether an individual gave their survey response before (within 1, 3, or 5 days) or after an attack (within 1, 3, or 5 days). We find that the treatment effect is indeed positive and statistically significant for most models. Substantively, the estimates suggest that individuals interviewed 3 or 5 days after a terrorist attack were, all else equal, 2–3 percentage points more likely to express that terrorism is an important issue. Note the strong consistency in the treatment effect estimates. A possible imbalance between groups is thus unlikely to have affected our identification strategy.

### Comparing Attack Effects

**Figure 3.**
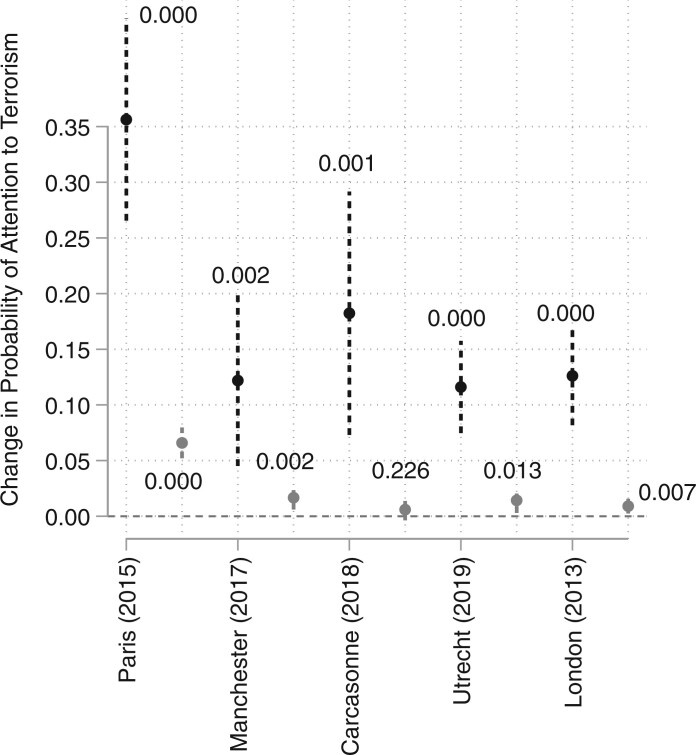
**Comparing effects across attacks.** Point estimates marked by dots, 95 percent confidence intervals marked by vertical bars. Black dots/lines: effect within targeted country; gray dots/lines: effect across Europe. The *p*-values are included next to each estimate, while attacks are ordered (left to right) by fatalities: Paris (2015): 137, Manchester (2017): 23, Carcassonne (2018): 5, Utrecht (2019): 3, London (2013): 1.

Our primary focus is the comparison of treatment effects across attacks. These analyses acknowledge that not all terrorist attacks are equal and, in fact, could well exert heterogeneous effects. Figure 3 displays the coefficient estimates and confidence intervals for all five attacks. The results are based on regressions that omit the controls and focus on before/after comparisons of 3 days. Estimates do not substantially change when including covariates or moving to 1 and 5 days before/after comparisons. Finally, we distinguish between effects across Europe and within the targeted country.

As figure 3 highlights, impacts within targeted states consistently dwarf effects across Europe. Still, the attention terrorism generates across Europe remains significant: no incident produced a negative effect, and all the incidents except Carcassonne 2018 have a statistically significant influence. In targeted countries, the audience was about 10–20 percentage points more likely to pay attention to terrorism. Across Europe, the impact of terrorist events on attention centers on 2–3 percentage points, which mirrors our results from above. However, the Bataclan attacks increased the likelihood that the French public saw terrorism as one of the two most important issues by more than 35 percentage points, and across Europe they had a strong effect with an increase of more than 6 points. Given the number of fatalities, it is not surprising that the substantively strongest effects are linked to Paris 2015. In sum, heterogeneity in the attacks’ effects does exist, and the number of fatalities is likely a strong influence, while there are important effect differences between the targeted country and a wider, cross-European audience.

### Comparing Attack-Size Effects

To shed more light on the presumed heterogeneity of incidents’ impact, we explore a potentially moderating effect stemming from fatalities. The findings for this disaggregation approach are based on similar regression models as for figure 2. We calculated models with and without controls for before/after comparisons of 1, 3, and 5 days for the full sample covering all countries. However, we now interact the treatment with the logged number of fatalities of each attack (logging the fatality count better accounts for the Paris outlier and the decreasing marginal effect of a single fatality on public opinion when the number of fatalities is large). Given that we cover a limited number of attacks, these results have suggestive character.

**Figure 4.**
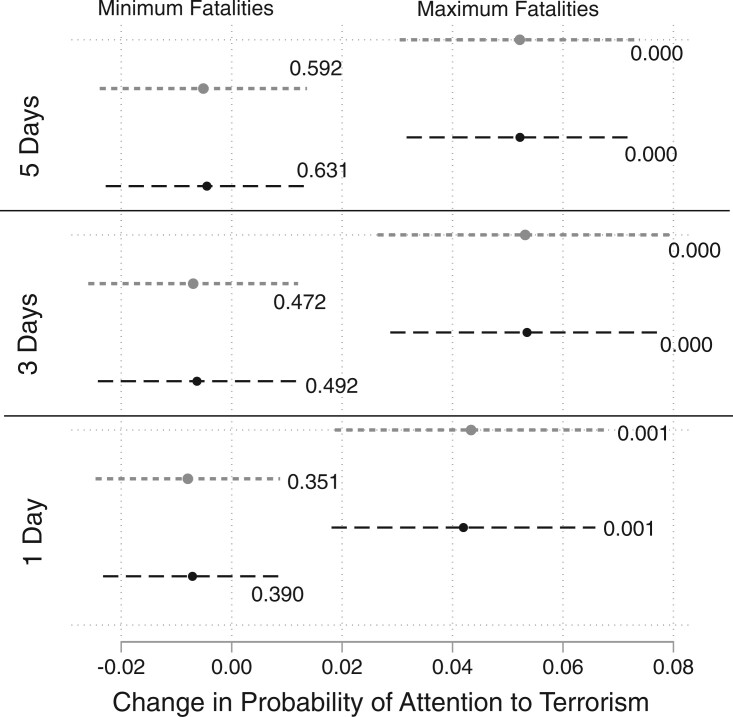
**Impact of attack size on *Attention to Terrorism***. Point estimates marked by dots, 95 percent confidence intervals marked by horizontal bars. Black dots/lines: models without controls; gray dots/lines: models with controls. Effects calculated for 1, 3, and 5 days before and after an attack. Left panel pertains to casualties set to minimum (1 fatality), right panel pertains to casualties set to maximum (137 fatalities). The *p*-values are included next to each estimate.

Figure 4 finds evidence for an interaction effect. The positive and significant treatment effect we identified in figure 2 persists for incidents with many fatalities, with a magnitude of around 4–5 percentage points. In other words, individuals interviewed after a high-fatality terrorist attack were, all else equal, about 4–5 percentage points more likely than people interviewed before to state that terrorism is an important issue affecting their country. Conversely, low-fatality terrorist attacks have little, if any, effect, given that the point estimates’ confidence intervals overlap with a value of 0 regardless of whether we look at before/after comparisons of 1, 3, or 5 days.

## Discussion

Terrorists intend to intimidate a broad audience to affect public opinion. Attention to terrorism is critical, as it represents a necessary condition through which terrorists impact political attitudes and governments’ reactions, but we still know little about whether terrorism succeeds in having a meaningful impact on public attention.

Our analysis combines the strengths of quasi-experiments in terms of internal validity with a comparative design to increase external validity. Specifically, we focus on five diverse terrorist incidents in the post-9/11 period and analyze people’s views expressed right before and after these attacks. Attacks do indeed causally, significantly, and substantively affect attention to terrorism. According to the pooled analysis, an attack increases the chances of people seeing terrorism as an important issue by more than 2 percentage points across Europe. In the targeted countries, effects are large, ranging from at least 11 percentage points for the Utrecht attack to 35 percentage points for Paris 2015. Even the relatively small attack in London in 2013 (one fatality) and Carcassonne 2018 have increased attention to terrorism by 13 percentage points in the UK and 19 points in France, respectively. The consequences of terrorism beyond a target state’s borders are statistically significant at conventional levels, but not very substantial. Still, while attacks have a more substantive impact at home than abroad, more lethal incidents are more likely to ensure that “terrorists get the attention they want.”

Our findings are in line with previous research on the newsworthiness of larger attacks (Sui et al. 2017), which induces the attention desired by terrorists (Rohner and Frey 2007; Jetter 2017). However, specific effects are driven by the type of media coverage and the ensuing political debates, which in turn depend on the proximity to an attack (Böhmelt, Bove, and Nussio 2019; Matthes, Schmuck, and von Sikorski 2019; Solheim 2021).

Our results demonstrate the public attention-grabbing nature of terrorism, but we still do not know from which other key concerns the attention is indeed displaced. Using the proposed research design, future research can address key questions such as how the intensity of coverage on one issue is influenced by the intensity of coverage on other issues and whether attention to terrorism contributes to the rise and fall of a specific topic. Given the media reporting of prominent attacks, this research also contributes to the long-lasting debate around the extent to which media salience changes first and public salience follows suit (e.g., Brosius and Kepplinger 1995; Geiß 2019).

Our study has some limitations. First, the limited number of cases prevents us from leveraging a richer heterogeneity in the lethality or intensity of attacks. Also, as only Islamist terrorist attacks satisfied our design criteria, we could not include other types of terrorist attacks, such as right-wing attacks, which have been the focus of recent media attention. While they may not modulate public opinion the same way Islamist attacks do, they could generate attention similarly (Huff and Kertzer 2018; Kearns, Betus, and Lemieux 2019). Future research should thus use a more diverse sample of attacks with variation in perpetrator identity, attack type, as well as number and type of victims. In this sense, analyzing five cases is only a first step to increasing the generalizability of findings based on a quasi-experimental approach.

Second, the limited time frame of analysis prevents us from identifying effects that go beyond the immediate aftermath of the attack. The duration of the effects is an important next frontier for this literature. It may be hard to study using the same design approach as we use here, given that the identifying assumptions of our quasi-experimental approach may only be valid for the time right around the attacks. Hence, future studies focusing on the durability of effects should use different methodological approaches. However, previous studies suggest that effects may in fact be limited to a few days after the attack (Legewie 2013; Nussio 2020).

## Data Availability Statement

REPLICATION DATA AND DOCUMENTATION are available at the Harvard Dataverse (https://doi.org/10.7910/DVN/DPWYDJ).

## Supplementary Material

SUPPLEMENTARY MATERIAL may be found in the online version of this article: https://doi.org/10.1093/poq/nfab046.

## Supplementary Material

nfab046_Supplementary_Data

## Footnotes

1 In total, we found 25 attacks covered in the GTD during a Eurobarometer survey period. However, only the five selected contained relevant questions, included sufficient sample sizes, and were known as terrorist attacks. The full list with all attacks covering half a million survey respondents is published in the Supplementary Material. Giani (2021) uses a similar approach based on the European Social Survey.

2 We do not include survey weights. Additional analyses with Eurobarometer weights did not change any of the results.

## Notes

The authors thank Aizhan Orozbakieva and Marta Lindström for excellent research assistance. They are also grateful to the editors of *POQ* and the three anonymous reviewers. This work was supported by the British Academy/Leverhulme Trust [SRG19\190780 to T.B].
