# Supplementary material for: Do Terrorists Get the Attention They Want? Comparing Effects of Terrorism across Europe
Source: Public Opin Q. 2021 Oct 7;85(3):900–12. doi: 10.1093/poq/nfab046 (PMC8643658; doi:10.1093/poq/nfab046)
Supplement: nfab046_Supplementary_Data [file nfab046_supplementary_data.pdf]

Do Terrorists Get the Attention They Want? Comparing Effects of Terrorism across Europe - Supplementary Material

Enzo Nussio
Center for Security Studies, ETH Zürich
Corresponding author
enzo.nussio@sipo.gess.ethz.ch

Tobias Böhmelt
University of Essex
Colchester, UK
tbohmelt@essex.ac.uk

Vincenzo Bove
University of Warwick
Coventry, UK
v.bove@warwick.ac.uk.

July 10, 2021

Do Terrorists Get the Attention They Want? Comparing Effects of Terrorism across Europe

This *Appendix* provides supplementary text and additional analyses that complement and further support the main article's argument and findings. These include:

- A **summary of the attacks** included in the main analysis.
- We present **further details on statistical analysis** and **tables comprising the results underlying the main text's figures**.
- The SI comprises **balance statistics** of the control variables (Table S8).
- We have conducted **an Oster Selection Test** to assess the sensitivity of our results (Figure S1).
- We present regular **logistic regression models** (Table S9).
- We assess whether there is a **diffusion effect** from terrorism abroad to **attention to terrorism at home** (Table S10).
- As a falsification test, we exchanged the outcome variable by **trust in the European Central Bank** and, as a placebo test, only **assign the treatment** to those **interviewed two days before an attack** (Table S11).
- We include individuals **interviewed on the day of an attack** (Table S12).
- In addition to fatalities, we also consider **wounded people** (Table S13).
- An overview of **all Eurobarometer surveys coinciding with terrorist attacks** until mid-2019 (Table S14).

Information on Attacks

In the following, we provide information on each of the nine attacks included in our data set.

- London, May 22, 2013: Two assailants hit Lee Rigby, a British Army soldier, with their car and then proceeded to attack the man with knives and a cleaver in London, United Kingdom. Rigby, an off-duty British soldier, was killed in the incident. Michael Adebolajo and Michael Adebowale claimed responsibility in a video shot at the scene and stated that the attack was carried out in retaliation for British soldiers killing Muslims abroad. This attack is covered by Eurobarometer round 79.3.
- Paris/Saint Denis, November 13, 2015: : Bataclan attack comprising eight coordinated Islamic State of Iraq and the Levant (ISIL) attacks in Paris/St. Denis, killing 137 people. Suicide bombers struck outside

the Stade de France (Saint-Denis), while several mass shootings and a suicide bombing occurred at coffee places and restaurants. This attack is covered by Eurobarometer round 84.3.

- Manchester, May 22, 2017: A suicide bomber, identified as Salman Abedi, detonated at the Manchester Arena following an Ariana Grande concert in Manchester, United Kingdom. In addition to Abedi, at least 22 people were killed and 119 people were injured in the blast. The Islamic State of Iraq and the Levant (ISIL) claimed responsibility and stated that the attack was carried out in response to "transgressions against the lands of the Muslims." This attack is covered by Eurobarometer round 87.3.

- Carcasonne, March 23, 2018: Redouane Lakdim stopped a car on the outskirts of Carcassonne and shot both of its occupants. Stealing the car, he then attacked four police officers by shooting at them and trying to run them over. In turn, he stormed a supermarket, killing two civilians and a police office, before the terrorist was killed by a police unit. In total, the attack saw five fatalities, including Lakdim, and 15 wounded. This attack is covered by Eurobarometer round 89.1.

- Utrecht, March 18, 2019: Three people were killed in a mass shooting on a tram in Utrecht, Netherlands (seven wounded people). Local police classified the incident as a terrorist attack. The suspect, a 37-year-old Turkish man, was arrested and he admitted carrying out the attack. The perpetrator, Gökmen Tanis, was charged with "murder with terrorist intent." This attack is covered by Eurobarometer round 91.2

Four additional attacks that occurred during the Eurobarometer survey period and were recorded by the GTD were not considered for analysis. We omitted three incidents classified as extreme right-wing terrorism ("National Socialist Underground") in Germany and the 2004 Madrid bombings. The German cases were not associated with terrorism at all, but rather crime, when committed; the Madrid bombings were excluded because the sample size for the analysis of the targeted country was too small to allow for robust inferences: only 107 people were interviewed in Spain before/after the incident, but only 9 of them within three days after the bombing.

Further Details on Statistical Analysis and Main Regression Models

In this section, we present further details on statistical analysis, including the main variables' descriptive statistics (Table S1), and the regression models underlying the graphs of the main text (Tables S2-S3). The Eurobarometer provides information on regional subdivisions and units at the NUTS1, NUTS2, or NUTS3 level, which divides the territory in relatively homogenous areas based on socioeconomic, cultural, and historical characteristics. The unit-level intercept is based on the lowest possible level of aggregation and is modeled according to a normal distribution. The survey-round intercept is based on the survey information in the Eurobarometer and also follows a normal distribution. In addition, these random intercepts control for the possible non-random selection of units that were surveyed first. While we believe this is not a major issue given the Eurobarometer's fieldwork approach and coding rules, the exact timing of fieldwork may vary across units.

Including random intercepts for NUTS units and survey rounds ensures that we carry out the pre and post comparisons within surveys and within units.

Regarding the controls summarized in Table S1 (the sample size is based on a 3 days before/after comparison) and used for most other analyses, we identified a series of relevant variables across several waves of the Eurobarometer that are operationalized in the same way, with the same question wordings. First, we control for respondents' age, which ranges between 15 and 99 in our sample (mean value of 49.82). Second, male respondents might differ from females in their views about terrorism. We created a variable, *Female*, which is coded 1 if a survey participant was female (0 otherwise). Third, when people indicated that they were not self-employed or employed, we code them as unemployed and include the corresponding binary variable in the analysis as well. Fourth, as urban interviewees could differ from individuals living in rural areas, we control for this with a dichotomous variable receiving a value of 1 if the latter scenario applies (0 otherwise). Finally, the variable *Education* captures individuals' overall degree of school/university education on a scale from 1 to 10.

Table S1. Descriptive Statistics

**Variable**	**Obs.**	**Mean**	**Std.Dev.**	**Min.**	**Max.**
Attention to Terrorism	83,769	0.080	0.272	0	1
Treatment	83,769	0.463	0.499	0	1
Fatalities (ln)	83,769	2.497	1.686	0.000	4.920
Age	83,764	49.816	18.208	15	99
Female	83,769	0.539	0.499	0	1
Rural	83,769	0.324	0.468	0	1
Unemployed	83,769	0.083	0.276	0	1
Education	81,001	5.863	2.813	1	10

Table S2. Attention to Terrorism: Main Models

	**Model 1**	**Model 2**	**Model 3**	**Model 4**	**Model 5**	**Model 6**
	**1 Day**	**1 Day**	**3 Days**	**3 Days**	**5 Days**	**5 Days**
Treatment	0.018	0.019	0.025**	0.024*	0.025**	0.025**
	(0.106)	(0.112)	(0.047)	(0.057)	(0.029)	(0.034)
Age		0.000***		0.000***		0.000***
		(0.004)		(0.004)		(0.007)
Female		0.006**		0.006**		0.006**
		(0.036)		(0.013)		(0.017)
Rural		0.000		−0.002		−0.002
		(0.926)		(0.692)		(0.531)
Unemployed		−0.011***		−0.013***		−0.009***
		(0.000)		(0.000)		(0.000)
Education		−0.003***		−0.002***		−0.002***
		(0.002)		(0.000)		(0.000)
Observations	30,657	29,676	83,769	80,999	113,421	109,689
Prob > χ^2	0.106	0.000	0.047	0.000	0.029	0.000

* $p < 0.10$,** $p < 0.05$,***$p < 0.01$
Robust standard errors are used and p-values in parentheses (two-tailed).
Constant included in all models, but omitted from presentation.
All models include random intercepts for units (NUTS level) and survey rounds (years).

Similar to the analyses in the first two tables, we also examined the effect of terrorism on public opinion in the focal country only (Tables S4-S5). That is, we omit all respondents not living in the country of an attack and re-estimate the main models. To provide a fully comprehensive overview of the effects, Tables S6 and S7 concentrate on the effects of an attack outside its borders only, i.e., we omit individuals from the target country. The main analyses pools respondents from a country under attack and from the wider European audience. As

Table S3. Attention to Terrorism: Interaction with *Fatalities (ln)*

	**Model 7** **1 Day**	**Model 8** **1 Day**	**Model 9** **3 Days**	**Model 10** **3 Days**	**Model 11** **5 Days**	**Model 12** **5 Days**
Treatment	−0.007	−0.008	−0.006	−0.007	−0.004	−0.005
	(0.390)	(0.351)	(0.492)	(0.472)	(0.631)	(0.592)
Fatalities (ln)	0.008	0.008	0.010	0.009	0.010	0.010
	(0.435)	(0.455)	(0.272)	(0.284)	(0.128)	(0.126)
Treatment × Fatalities (ln)	0.010***	0.010***	0.012***	0.012***	0.012***	0.012***
	(0.003)	(0.002)	(0.001)	(0.002)	(0.001)	(0.001)
Age		0.000***		0.000***		0.000***
		(0.004)		(0.004)		(0.008)
Female		0.006**		0.006**		0.006**
		(0.047)		(0.013)		(0.018)
Rural		0.000		−0.001		−0.002
		(0.948)		(0.691)		(0.482)
Unemployed		−0.010***		−0.013***		−0.009***
		(0.000)		(0.000)		(0.000)
Education		−0.003***		−0.002***		−0.002***
		(0.001)		(0.000)		(0.000)
Observations	30,657	29,676	83,769	80,999	113,421	109,689
Prob > χ^2	0.000	0.000	0.000	0.000	0.000	0.000

* $p < 0.10$,** $p < 0.05$,***$p < 0.01$
Robust standard errors are used and p-values in parentheses (two-tailed).
Constant included in all models, but omitted from presentation.
All models include random intercepts for units (NUTS level) and survey rounds (years).

expected, the core finding is robust, but more strongly pronounced when focusing on the target state. Hence, proximity matters and terrorism more closely to a respondent has a bigger effect than more distant attacks (see also Böhmelt, Bove and Nussio, 2020; Nussio, Bove and Steele, 2019). And incidents in one's own state remain to be the most proximate events one could think of.

Table S4. Attention to Terrorism: Main Models for Target Country Only

	**Model 13** **1 Day**	**Model 14** **1 Day**	**Model 15** **3 Days**	**Model 16** **3 Days**	**Model 17** **5 Days**	**Model 18** **5 Days**
Treatment	0.205**	0.213**	0.161***	0.162***	0.171***	0.173***
	(0.017)	(0.014)	(0.000)	(0.000)	(0.000)	(0.000)
Age		−0.001		−0.001		−0.001*
		(0.484)		(0.372)		(0.100)
Female		0.095***		0.054***		0.050***
		(0.007)		(0.002)		(0.000)
Rural		−0.008		−0.015		−0.018
		(0.754)		(0.575)		(0.490)
Unemployed		−0.084**		−0.062*		−0.059***
		(0.022)		(0.055)		(0.010)
Education		−0.014**		−0.017***		−0.018***
		(0.031)		(0.000)		(0.000)
Observations	936	923	2,686	2,653	3,623	3,576
Prob > χ^2	0.017	0.000	0.000	0.000	0.000	0.000

* $p < 0.10$,** $p < 0.05$,***$p < 0.01$
Robust standard errors are used and p-values in parentheses (two-tailed).
Constant included in all models, but omitted from presentation.
All models include random intercepts for units (NUTS level) and survey rounds (years).

Balance Statistics

The timing of attacks must be exogenous relative to that of the Eurobarometer interviews in order to meet a key identification requirement of our causal-estimation approach. It is plausible to assume that this is given in our setup. That said, there are other potential sources of bias. Specifically, individuals with specific features

Table S5. Attention to Terrorism: Interaction with *Fatalities (ln)* for Target Country Only

	**Model 19**<br>**1 Day**	**Model 20**<br>**1 Day**	**Model 21**<br>**3 Days**	**Model 22**<br>**3 Days**	**Model 23**<br>**5 Days**	**Model 24**<br>**5 Days**
Treatment	0.027	0.046	0.083***	0.081**	0.090***	0.091***
	(0.650)	(0.495)	(0.007)	(0.023)	(0.002)	(0.009)
Fatalities (ln)	0.023	0.023	0.032	0.031	0.030	0.029
	(0.549)	(0.557)	(0.182)	(0.195)	(0.135)	(0.161)
Treatment × Fatalities (ln)	0.064***	0.060***	0.039**	0.040*	0.043***	0.043***
	(0.000)	(0.004)	(0.050)	(0.062)	(0.004)	(0.010)
Age		−0.001		−0.001		−0.001
		(0.492)		(0.349)		(0.111)
Female		0.088**		0.053***		0.049***
		(0.023)		(0.004)		(0.000)
Rural		−0.011		−0.015		−0.019
		(0.663)		(0.543)		(0.456)
Unemployed		−0.085**		−0.062*		−0.059***
		(0.018)		(0.051)		(0.009)
Education		−0.013**		−0.017***		−0.018***
		(0.028)		(0.000)		(0.000)
Observations	936	923	2,686	2,653	3,623	3,576
Prob > χ^2	0.000	0.000	0.000	0.000	0.000	0.000

* $p < 0.10$,** $p < 0.05$,***$p < 0.01$
Robust standard errors are used and p-values in parentheses (two-tailed).
Constant included in all models, but omitted from presentation.
All models include random intercepts for units (NUTS level) and survey rounds (years).

Table S6. Attention to Terrorism: Main Models for Cross-Europe Only

	**Model 25**<br>**1 Day**	**Model 26**<br>**1 Day**	**Model 27**<br>**3 Days**	**Model 28**<br>**3 Days**	**Model 29**<br>**5 Days**	**Model 30**<br>**5 Days**
Treatment	0.014*	0.015*	0.021*	0.021*	0.021*	0.021*
	(0.094)	(0.099)	(0.072)	(0.084)	(0.050)	(0.055)
Age		0.000**		0.000**		0.000
		(0.019)		(0.042)		(0.378)
Female		0.003		0.004**		0.004**
		(0.283)		(0.041)		(0.033)
Rural		0.000		0.001		−0.001
		(0.928)		(0.874)		(0.772)
Unemployed		−0.009***		−0.012***		−0.007***
		(0.000)		(0.000)		(0.000)
Education		−0.002***		−0.002***		−0.001***
		(0.001)		(0.000)		(0.001)
Observations	30,823	29,855	84,441	81,704	114,978	11,293
Prob > χ^2	0.009	0.000	0.072	0.000	0.050	0.000

* $p < 0.10$,** $p < 0.05$,***$p < 0.01$
Robust standard errors are used and p-values in parentheses (two-tailed).
Constant included in all models, but omitted from presentation.
All models include random intercepts for units (NUTS level) and survey rounds (years).

Table S7. Attention to Terrorism: Interaction with *Fatalities (ln)* for Cross-Europe Only

	Model 31<br>1 Day	Model 32<br>1 Day	Model 33<br>3 Days	Model 34<br>3 Days	Model 35<br>5 Days	Model 36<br>5 Days
Treatment	−0.007	−0.008	−0.010	−0.011	−0.009	−0.010
	(0.264)	(0.225)	(0.217)	(0.206)	(0.273)	(0.240)
Fatalities (ln)	0.004	0.004	0.005	0.004	0.005	0.005
	(0.408)	(0.464)	(0.336)	(0.370)	(0.208)	(0.211)
Treatment × Fatalities (ln)	0.008***	0.008***	0.012***	0.012***	0.011***	0.011***
	(0.000)	(0.000)	(0.000)	(0.001)	(0.000)	(0.000)
Age		0.000**		0.000*		0.000
		(0.019)		(0.050)		(0.390)
Female		0.003		0.004**		0.004**
		(0.304)		(0.039)		(0.034)
Rural		0.000		0.001		−0.001
		(0.903)		(0.849)		(0.742)
Unemployed		−0.009***		−0.012***		−0.007***
		(0.001)		(0.000)		(0.000)
Education		−0.002***		−0.002***		−0.001***
		(0.001)		(0.000)		(0.001)
Observations	30,823	29,855	84,441	81,704	114,978	11,293
Prob > χ^2	0.000	0.000	0.000	0.000	0.000	0.000

* $p < 0.10$,** $p < 0.05$,***$p < 0.01$
Robust standard errors are used and p-values in parentheses (two-tailed).
Constant included in all models, but omitted from presentation.
All models include random intercepts for units (NUTS level) and survey rounds (years).

may be more likely to be interviewed than others. If these characteristics correlate with the outcome variable, too, our results may be biased. In Table S6, we assess whether there are systematic differences in our control variables across individuals interviewed before and after an attack using normalized differences: Imbens and Wooldridge (2009) suggest as a rule of thumb that with a normalized difference exceeding one quarter, linear regression is likely to be sensitive to model specifications. However, as Table S8 shows, this is unlikely to be an issue in our case.

Table S8. Normalized Differences

**Variable**	**Estimation**
Age	-0.019
Female	0.009
Rural	-0.007
Unemployed	-0.027
Education	0.015

Table entries are normalized differences following Imbens and Wooldridge (2009).

When examining the patterns in missing data and non-responses, most of those cases do not pertain to a specific question, but all items of the Eurobarometer. There is also little support for the claim that missing data or non-responses in the outcome variable are more likely among those interviewed after an attack.

Oster Selection Test

In light of Balcells and Torrats-Espinosa (2018), we also assess the sensitivity of our results using one of Oster's selection tests (Oster, 2019). While we are confident in the validity of our research design's assumptions, a last test in this context examines the level of unobserved confounding that would make the results become invalid. Specifically, given a specific R^2 value (in essence, the one reported for our main, unconditional model

that includes the control variables and is based on before/after comparisons of 3 days), we vary the degree of unobserved selection into treatment, which is defined as the ratio of selection on unobservables to selection on observables and calculate how the treatment effect would change. In substantive terms, a ratio of selection on unobservables to selection on observables of 1 implies that the unobservables are as important as the observables. However, as Figure S1 highlights, when keeping the R^2 value constant, altering the ratio does not fundamentally change the size of the treatment effect.

Figure S1. Oster Selection Test

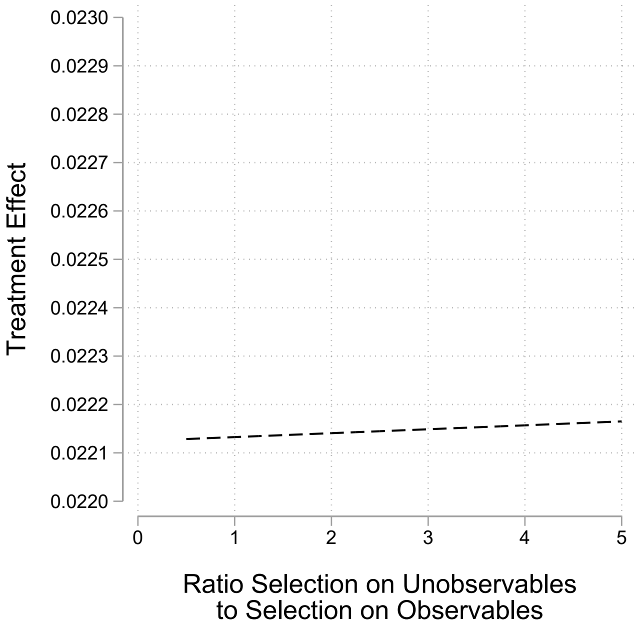

Logistic Regression Models

The hierarchical models we employ are linear models, rendering all estimations essentially linear-probability models that control for the hierarchical nature of the data. A key advantage of this setup is that coefficients can be directly interpreted. However, a potential shortcoming is that we induce heteroskedasticity when employing a linear model on a binary dependent variable. To address any concerns in this regard, we re-estimated the main (unconditional) models with regular logistic regression models that comprise fixed effects for survey rounds and NUTS units. In this setup, only the signs of the coefficients and their significance levels can be interpreted directly. However, as Table S9 emphasizes, our findings are robust to the change in the estimator.

Diffusion Effect

The main finding in Böhmelt, Bove and Nussio (2020) suggests that terrorism abroad can shape migration attitudes at home. That is, not only attacks in the focal country shape public opinion, but also incidents in other countries. By implication, one may also expect a relatively strong impact, e.g., of terrorist attacks in

Table S9. Attention to Terrorism: Logistic Regression Models

	**Model 37**	**Model 38**
Treatment	0.408***	−0.198***
	(0.000)	(0.002)
Fatalities (ln)		0.899***
		(0.000)
Treatment × Fatalities (ln)		0.210***
		(0.000)
Age	−0.002**	−0.002**
	(0.048)	(0.038)
Female	0.107***	0.107***
	(0.000)	(0.000)
Rural	−0.006	−0.005
	(0.850)	(0.882)
Unemployed	−0.218***	−0.218***
	(0.000)	(0.000)
Education	−0.030***	−0.031***
	(0.000)	(0.000)
Observations	77,601	77,601
Prob > χ^2	0.000	0.000

* $p < 0.10$,** $p < 0.05$,***$p < 0.01$

Robust standard errors are used and p-values in parentheses (two-tailed).

Constant included in all models, but omitted from presentation.

Models include unit and survey fixed effects.

France on public opinion in Germany. Similar patterns seem plausible for the UK and Ireland, or the Netherlands and Belgium. To explore this possibility in more detail, Tables S6 and S7 above examine a cross-European only effect, but we created here a variable that captures whether the country of a terrorist attack and the state where an individual has been interviewed are neighbors as defined in Stinnett et al. (2002). To code country neighbors, we focus on direct land borders and separation of up to 24 miles of water. We interact this contiguity variable with the treatment and re-estimate our core model with the control covariates.

According to Table S10 in this appendix, the main result is also robust to considering a diffusion effect of terrorism on public opinion, but we do indeed find evidence for the latter as well (also similar to Tables S6 and S7). On one hand, the treatment's coefficient now pertains to those cases where *Contiguity* is set to 0, i.e., we omit neighboring countries here. The coefficient estimate remains statistically significant and positively signed in the first column, providing further evidence for a causal impact of terrorism at home on public opinion. On the other hand, the interactive term's coefficient captures those cases where public opinion is influenced by terrorism in the neighborhood – and the estimate is positive and significant as well at least in Model 39. This finding may add to the "European sphere" literature (e.g., Trenz, 2005).

Falsification and Placebo Tests

To further increase the confidence in our findings, we conducted one falsification test and, secondly, assigned a placebo treatment at a different date than the actual terrorist attacks (see also Muñoz, Falcó-Gimeno and Hernández, 2020). On one hand, we replaced the outcome variable by an item, which should substantively not

Table S10. Attention to Terrorism: Diffusion Effect

	**Model 39**	**Model 40**
Treatment	0.020*	−0.007
	(0.069)	(0.470)
Contiguity	0.016	0.014
	(0.424)	(0.562)
Treatment × Contiguity	0.037**	0.007
	(0.030)	(0.779)
Fatalities (ln)		0.008
		(0.246)
Treatment × Fatalities (ln)		0.011***
		(0.006)
Contiguity × Fatalities (ln)		0.002
		(0.788)
Treatment × Contiguity × Fatalities (ln)		0.009
		(0.129)
Age	0.000***	0.000***
	(0.003)	(0.004)
Female	0.006**	0.006**
	(0.012)	(0.014)
Rural	−0.001	−0.001
	(0.706)	(0.708)
Unemployed	−0.013***	−0.013***
	(0.000)	(0.000)
Education	−0.002***	−0.002***
	(0.000)	(0.000)
Observations	80,999	80,999
Prob > χ^2	0.000	0.000

* $p < 0.10$,** $p < 0.05$,***$p < 0.01$

Robust standard errors are used and p-values in parentheses (two-tailed).

Constant included in all models, but omitted from presentation.

Models include unit and survey fixed effects.

be affected by terrorist attacks. Instead, the substantive meaning of this variable makes it virtually impossible to identify a plausible relationship with the treatment. This implies that if we were to find a statistically significant relationship with our treatment and the new dependent variable having these characteristics, we may simply pick up a spurious relationship. For this test, we have opted for a Eurobarometer item on trust in the European Central Bank (ECB). Interviewees were asked what institutions they generally trust, and the ECB is among the possible answers. We used this information to create a binary variable receiving a value of 1 if individuals answered that they trust in the ECB institution and 0 otherwise. In turn, we use this item as the new dependent variable and re-run the main, unconditional model based on before/after comparisons of 3 days). On the other hand, we altered the treatment by assigning it only to people interviewed two days before an attack took place. To this end, we follow Muñoz, Falcó-Gimeno and Hernández (2020, p.12) as we "address the possibility that preexisting time trends, unrelated to the event of interest, could bias the findings," and thus "test for the existence of such a trend before the event took place." Muñoz, Falcó-Gimeno and Hernández (2020, p.12) suggest "placebo treatments constructed at arbitrary points at the left of the cutoff-point," and two days before an attack meets this criterion. It seems implausible that those interviewees would know about an incident to come and, hence, we should not detect a significant treatment effect either. Table S11 summarizes the corresponding results.

Table S11. Attention to Terrorism: Falsification and Placebo Tests

	**Model 41**<br>**ECB Trust**	**Model 42**<br>**ECB Trust**	**Model 43**<br>**2 Days Prior Attack**	**Model 44**<br>**2 Days Prior Attack**
Treatment	−0.002	0.004	−0.010	0.010
	(0.559)	(0.242)	(0.408)	(0.361)
Fatalities (ln)		−0.005		0.017***
		(0.489)		(0.001)
Treatment × Fatalities (ln)		−0.002*		−0.008
		(0.062)		(0.113)
Age	0.000	0.000	0.000***	0.000***
	(0.234)	(0.231)	(0.003)	(0.003)
Female	−0.020**	−0.020**	0.006**	0.006***
	(0.016)	(0.016)	(0.011)	(0.010)
Rural	−0.023***	−0.024***	−0.002	−0.001
	(0.000)	(0.000)	(0.703)	(0.757)
Unemployed	−0.088***	−0.088***	−0.013***	−0.013***
	(0.000)	(0.000)	(0.000)	(0.000)
Education	0.024***	0.024***	−0.002***	−0.002***
	(0.000)	(0.000)	(0.000)	(0.000)
Observations	50,517	50,517	80,999	80,999
Prob > χ^2	0.000	0.000	0.000	0.000

* $p < 0.10$,** $p < 0.05$,***$p < 0.01$

Robust standard errors are used and p-values in parentheses (two-tailed).

Constant included in all models, but omitted from presentation.

All models include random intercepts for units (NUTS level) and survey rounds (years).

The treatment effect is statistically insignificant in all models, while the interaction with the fatality variable is, in fact, negative in Model 42. The results associated with the other controls seem reasonable and/or unchanged across all estimations when compared with the main model. In sum, this further increases our

confidence in the validity of the main result.

Inclusion of People Interviewed on the Day of an Attack

In all previous models and the ones presented in the main text, we exclude individuals who were interviewed on the day of an attack. On one hand, we cannot fully ensure that an interview then took place indeed after an incident and not before. On the other hand, even if an interview, say, was conducted about two hours after a terrorism attack, it may not have made it to the news and is covered by the media so that individuals can be affected by this. However, as a robustness check, we do include all people interviewed on the day of an attack and, as demonstrated, this does not affect the substance of our main result (Table S12).

Table S12. Attention to Terrorism: Inclusion of People Interviewed on the Day of an Attack

	**Model 45**	**Model 46**
Treatment	0.019**	−0.001
	(0.015)	(0.819)
Fatalities (ln)		0.008
		(0.304)
Treatment × Fatalities (ln)		0.008***
		(0.000)
Age	0.000***	0.000***
	(0.007)	(0.008)
Female	0.005**	0.005**
	(0.024)	(0.025)
Rural	−0.003	−0.003
	(0.371)	(0.370)
Unemployed	−0.013***	−0.013***
	(0.000)	(0.000)
Education	−0.002***	−0.002***
	(0.000)	(0.000)
Observations	96,042	96,042
Prob > χ^2	0.000	0.000

* $p < 0.10$,** $p < 0.05$,***$p < 0.01$
Robust standard errors are used and p-values in parentheses (two-tailed).
Constant included in all models, but omitted from presentation.
Models include unit and survey fixed effects.

Inclusion of Wounded People

While we have considered the influence of fatalities, our model estimations did not include the number of wounded people. However, a large number of wounded may also affect media attention and, eventually, public opinion. Against this background, we created the variable *Casualties (ln)*, which is the logarithm of the number of dead and wounded casualties of an attach. Table S13 summarizes our findings for models with controls for before/after comparisons of 1, 3, and 5 days for the full sample covering all countries. When additionally considering wounded victims, however, the results are virtually identical to those merely focusing on fatalities only.

Table S13. Attention to Terrorism: Interaction with *Casualties (ln)*

	**Model 47** **1 Day**	**Model 48** **3 Days**	**Model 49** **5 Days**
Treatment	−0.009	−0.009	−0.007
	(0.394)	(0.529)	(0.623)
Casualties (ln)	0.009	0.009	0.009**
	(0.271)	(0.137)	(0.038)
Treatment × Casualties (ln)	0.007**	0.009**	0.008**
	(0.042)	(0.049)	(0.037)
Age	0.000***	0.000***	0.000***
	(0.003)	(0.004)	(0.008)
Female	0.006**	0.006**	0.006**
	(0.046)	(0.014)	(0.018)
Rural	0.000	−0.001	−0.002
	(0.948)	(0.700)	(0.493)
Unemployed	−0.011***	−0.013***	−0.009***
	(0.000)	(0.000)	(0.000)
Education	−0.003***	−0.002***	−0.002***
	(0.001)	(0.000)	(0.000)
Observations	29,676	80,999	109,689
Prob > χ^2	0.000	0.000	0.000

* $p < 0.10$,** $p < 0.05$,***$p < 0.01$

Robust standard errors are used and p-values in parentheses (two-tailed).

Constant included in all models, but omitted from presentation.

All models include random intercepts for units (NUTS level) and survey rounds (years).

Table S14. Eurobarometer Surveys Coinciding with Terrorism

**EB-Round**	**Survey ID**	**Date of Attack**	**Country**	**City**	**Fatalities**	**Injuries**	**Type**
32	1752	1989-10-28	PT	Lisbon	1	0	far-right
36	2081	1991-11-02	GR	Athens	1	8	far-left
37.2	2242	1992-04-30	ES	Madrid	2	0	far-left
38.1	2295	1992-11-23	GER	Moelln	3	0	far-right
41.1	2491	1994-07-04	GR	Athens	1	0	far-left
53	3296	2000-05-08	ES	Vigo	2	4	far-left
54.1	3387	2000-11-17	ES	Madrid	1	0	far-left
55.2	3509	2001-06-13	GER	Nuremberg	1	0	far-right
56	3625	2001-08-29	GER	Munich	1	0	far-right
61	4056	2004-03-11	ES	Madrid	195	1800	Islamist
63.4	4411	2005-06-09	GER	Nuremberg	1	0	far-right
65.2	4506	2006-04-04	GER	Dortmund	1	0	far-right
67.2	4530	2007-04-25	GER	Heilbronn	1	1	far-right
71.3	4973	2009-06-17	GR	Athens	1	0	far-left
75.1	5479	2011-03-02	GER	Frankfurt	2	2	Islamist
77.2	5598	2012-03-19	FR	Toulouse	4	0	Islamist
79.3	5689	2013-05-22	UK	London	1	0	Islamist
84.2	6642	2015-10-22	SE	Trollhattan	4	1	far-right
84.3	6643	2015-11-13	FR	Paris and Saint Denis	137	413	Islamist
87.1	6861	2017-03-23	UK	London	6	50	Islamist
87.2	6862	2017-04-20	FR	Paris	2	3	Islamist
87.3	6863	2017-05-22	UK	Manchester	23	119	Islamist
87.4	6924	2017-06-19	UK	London	1	12	far-right
89.1	6963	2018-03-23	FR	Carcasonne	5	15	Islamist
91.2	7562	2019-03-18	NL	Utrecht	3	7	Islamist

All Eurobarometer Surveys Coinciding with Terrorism

Although we focus on Islamic terrorism, we also identified a series of other incidents that coincide with Eurobarometer surveys. Table S14 summarizes the necessary information. The information on attacks was retrieved from the Global Terrorism Database.[1] Most of these surveys were not used for our analysis as they did not include the relevant survey questions (e.g., "what do you think are the two most important issues facing (OUR COUNTRY) at the moment?").

References

Balcells, Laia and Gerard Torrats-Espinosa. 2018. "Using a natural experiment to estimate the electoral consequences of terrorist attacks." *Proceedings of the National Academy of Sciences* 115(42):10624–10629.

Böhmelt, Tobias, Vincenzo Bove and Enzo Nussio. 2020. "Can Terrorism Abroad Influence Migration Attitudes at Home?" *American Journal of Political Science* 64(3):437–457.

Imbens, Guido and Jeffrey Wooldridge. 2009. "Recent developments in the econometrics of program evaluation." *Journal of Economic Literature* 47(1):5–86.

Muñoz, Jordi, Albert Falcó-Gimeno and Enrique Hernández. 2020. "Unexpected Event During Surveys Design: Promise and Pitfalls." *Political Analysis* 28(2):186–206.

Nussio, Enzo, Vincenzo Bove and Bridget Steele. 2019. "The consequences of terrorism on migration attitudes across Europe." *Political Geography* 75(102047):1–10.

Oster, Emily. 2019. "Unobservable selection and coefficient stability: Theory and evidence." *Journal of Business & Economic Statistics* 37(2):187–204.

Stinnett, Douglas M, Jaroslav Tir, Paul F Diehl, Philip Schafer and Charles Gochman. 2002. "The Correlates of War (COW) project direct contiguity data, version 3.0." *Conflict Management and Peace Science* 19(2):59–67.

Trenz, Hans-Jörg. 2005. "The European Public Sphere: Contradictory Findings in a Diverse Research Field." *European Political Science* 4(4):407–420.

[1] Available at: `https://www.start.umd.edu/data-tools/global-terrorism-database-gtd`.
